# Improving annotation, access, and comparison of nutritional composition data for underutilized crops

**DOI:** 10.1093/database/baag050

**Published:** 2026-08-25

**Authors:** Agnes Aboagye, Paul D Shaw, Sebastian Raubach, Sean Mayes, Graham King, Guillermina M Mendiondo

**Affiliations:** Division of Plant and Crop Sciences, The University of Nottingham, Leicestershire LE12 5RD, United Kingdom; Department of Information and Computational Sciences, The James Hutton Institute, Invergowrie, DD2 5DA Dundee, United Kingdom; Department of Information and Computational Sciences, The James Hutton Institute, Invergowrie, DD2 5DA Dundee, United Kingdom; Division of Plant and Crop Sciences, The University of Nottingham, Leicestershire LE12 5RD, United Kingdom; Southern Cross University, Lismore, NSW 2480, Australia; Division of Plant and Crop Sciences, The University of Nottingham, Leicestershire LE12 5RD, United Kingdom

## Abstract

Open and interoperable data infrastructures are essential to advancing food and nutritional security research, yet few data management systems are specifically designed for crop nutritional datasets that support seamless querying, visualization, and comparison. Existing data sources are often syntactically and semantically heterogeneous, creating substantial barriers to interoperability and data reuse, and ultimately limiting the translation of research into sustainable agricultural innovation. To address this gap, we developed an ontology-based data access and integration (OBDI) framework that harmonizes publicly available, heterogeneous plant nutritional datasets within a unified semantic structure. By annotating datasets using established plant science ontologies, we enhanced their Findability, Accessibility, Interoperability, and Reusability by providing a scalable mechanism for virtual data integration via a knowledge graph enriched with domain semantics. Our workflow enables consistent comparison and computational reasoning across crop nutritional composition data, supporting both machine and human interpretation. The integration of compositional datasets for underutilized crops (UCs) alongside major crops allows for the identification of genetic resources that provide enhanced nutritional outcomes, fostering evidence-based diversification strategies. We outline how this approach may facilitate data integration and assist in the wider adoption and utilization of UCs. More generally, this open, ontology-driven approach highlights how investment in standardized, FAIR-aligned data infrastructure has the potential to accelerate interdisciplinary collaboration across plant sciences, nutrition, and policy. We demonstrate the concepts and specific methodologies for establishing semantic relationship between diverse datasets useful in operationalizing heterogeneous crop trait phenotyping knowledge bases. Furthermore, we discuss how the integration of ontology-based data integration (OBDI) with machine learning enables intelligent, accessible trait data management, thereby enhancing the adoption and advancement of AI-driven decision-making.

## Introduction

### Underutilized crops for food and nutrition security

Underutilized crops (UCs) are domesticated plants that are adapted to local environments and used as traditional food sources. However, they have seen limited adoption by farmers and have yet to be integrated into formal breeding programmes, which is likely due to a combination of factors, including their generally lower yields, restricted market demand, and particular agronomic challenges, such as vulnerability to pests or sensitivity to specific climatic conditions. These limitations have contributed to their comparatively limited research attention relative to major staple crops [1, 2]. Also referred to as orphan or marginal crops, UCs often retain sufficient genetic diversity to remain climate resilient and thus adapted to changing environmental conditions, with some being regarded as nutritionally dense [3, 4]. UCs, therefore, represent a significant untapped genetic repository that has the potential to be more widely used to help tackle the challenges of food and nutrition security in response to climate change [5]. Characterization and evaluation data for these crops are often poorly documented, reducing the opportunity for wider adoption and utilization. Although the major grains, including rice (*Oryza sativa L*.), wheat (*Triticum aestivum L*.), maize (*Zea mays L*.), and barley (*Hordeum vulgare L*.), contribute to the majority of calorific intake in humans, these crops do not have optimal concentrations of amino acids, minerals, vitamins, and protective phytochemicals essential for healthy growth, development, and wellbeing [6, 7]. Reliance on the consumption of these starch-rich cereals contributes to malnutrition, which is evident in areas where they represent the main source of calories [3]. In contrast, a crop such as Bambara groundnut (*Vigna subterranea* (L.) Verdc.) is an exemplar of an underutilized African legume, which has been termed a ‘complete’ food because of its balanced composition of proteins, carbohydrates, unsaturated fats, and essential nutrients [7].

### Nutritional data management systems for UCs

When compared to other crops that have benefited from research and policy support leading to rapid expansion of adoption and cultivation, UCs receive less research attention and investment [6, 8]. Few data management systems are available that enable systematic comparison and sharing of nutritional composition trait datasets either within or between crops. Inconsistent and inadequate data management is apparent not only for UCs, but also for major and minor crops [3, 9]. These limitations can also hinder the systematic evaluation of genetic diversity collections and pre-breeding research characterization, both of which are critical for developing resilient, well-adapted cropping systems. Specific challenges hindering data integration from different sources include inconsistencies in the measurement of traits, data collection, collation, storage, maintenance, data formats, and inadequate curation practices [10–12]. Many pre-breeding, genetic resource collections (such as those held in genebanks), and systematic breeding evaluation programmes are limited in their ability to allocate resources to data management and typically maintain and/or publish their data in spreadsheets. While spreadsheets are straightforward and convenient to manage and use, they have drawbacks that make them difficult to maintain and manage, limiting the interoperability and reusability of the data they contain [10]. CropBASE (https://cropbase.co.uk/cropbasev5/) is an extensive knowledge base providing information on UCs, including nutritional composition data, to support farmers and researchers in exploring, researching, and cultivating UCs. Germinate (https://germinate.hutton.ac.uk [12, 13]) is a general-purpose plant genetic resource database that provides tools for managing collection-related information, as well as plant accession passport data, phenotypic, genotypic, image, and field trial data from plant genetic resources collections. CropBASE and Germinate both hold different, but complementary data types. Germinate has a deeper scope within individual crops, especially focusing on genomics and breeding, while CropBASE has a wider scope across crop-level diversity with platforms including the CropDB database that collates information on the agroecological requirements, nutritional value, and potential applications of a wide range of UCs. While both CropBASE and Germinate serve different purposes, they have in common the management of heterogeneous and complementary data that have the potential to be semantically integrated and thus provide new data resources for UCs. Datasets describing variations in the nutritional composition of UCs are important in connecting dietary nutrition requirements with crop genetic improvement [14, 15]. Nutrient concentrations published in food composition databases provide a standardized representation of the nutrient profile for crops at the species or commodity level. Generally, these aggregate values are important for informing public health nutrition, dietary guidelines, and food policy [16]. Hence, modelling these nutritional variations between crops can inform breeding programmes, targeted nutrition strategies, and precision agriculture [17, 18]. Data interoperability and reuse are important requirements within the Findable, Accessible, Interoperable, and Reusable (FAIR) guiding principles that promote knowledge discovery and representation [19]. Thus, making crop-specific nutritional information available in databases alongside data exploration, analysis, and visualization tools should help simplify comparison between competing alternative crops and reduce the barriers to research data sharing [20, 21].

### Ontologies for data integration and interoperability

The use of ontologies substantially enhances phenotypic database management systems [22–24] by providing formal, controlled vocabularies with explicitly defined relationships between classes and concepts. Ontologies are formal models that explicitly define classes (terms) and concepts with controlled vocabularies, organized so that relationships between classes are explicitly and unambiguously defined [25]. The Open Biomedical and Biological Foundry (OBO; https://obofoundry.org) develops and harmonizes ontologies based on formal principles to support consistent interpretation across datasets [26].

The Planteome initiative (https://planteome.org/) provides a suite of reference ontologies for the plant sciences, including Plant Ontology (PO), Plant Trait Ontology (TO), and Plant Stress Ontology, which support model and crop-specific databases and enable cross-species trait searches, facilitating comparative analyses [19, 27, 28]. Other relevant ontologies include Chemical Entities of Biological Interest (ChEBI), an OBO ontology that provides a structured vocabulary of molecular entities relevant to organisms [29], and Food Ontology (FoodOn), which covers raw food sources, ingredients, and process terms for packaging, cooking, and preservation, derived from livestock, fish, and other organic and inorganic sources [30]. The Compositional Dietary Nutritional Ontology (CDNO), which precisely describe molecular, food source, and nutritional data, respectively [9].

Annotating data with ontology terms provide precise and shared meaning, which is a fundamental component of the FAIR data principles and necessary for enhancing data interoperability and effective stewardship [9, 19]. Despite this, managing diverse datasets, particularly in plant science, continues to present significant data integration and interoperability challenges. Expressing ontologies as controlled vocabulary, using formal ontology languages like Web Ontology Language (OWL) and Resource Description Framework (RDF), provides a robust, widely used and standardized framework that supports semantic consistency and enables seamless integration of diverse data sources [22, 31, 32]. These World Wide Web Consortium (W3C) standards enable reasoning and knowledge inference, with data queried and manipulated via the SPARQL language (https://www.w3.org/TR/sparql11-query/), which operates on a mathematical graph structure to enable semantic inferences, offering an innovative approach to data management that can significantly enhance representation for downstream analysis [21]. The https://planteome.org/ provides a suite of reference ontologies, which support model- and crop-specific databases and enable trait-specific searches across multiple species without requiring users to specify individual species, facilitating cross-species comparative analysis [19, 27, 28]. ChEBI is an OBO ontology providing a structured vocabulary of molecular entities relevant to organisms [29], while FoodOn covers raw food sources, ingredients, and process terms for packaging, cooking, and preservation across livestock, fish, and other organic and inorganic sources [30]. The CDNO provides structured terminologies to describe nutritional components and their concentrations contributing to the human diet [9]. Data integration and interoperability remain significant challenges in managing diverse datasets, particularly in plant science. OWL and RDF are W3C semantic web technology standards that enable access to, facilitate understanding and reasoning with, and infer new knowledge from data. Information stored in OWL and RDF formats can be queried, updated, and retrieved using the RDF-based SPARQL query language (https://www.w3.org/TR/sparql11-query/). SPARQL facilitates the retrieval, updating, and modification of information in a mathematical graph format. This graph-oriented approach enables semantic inferences, offering a valuable and innovative means of data management and, as a result, can significantly enhance the representation of data for downstream analysis and subsequent use [21].

### Ontology-based data access and integration

Ontology-based data access and integration (OBDI) provides a structured approach for data access, integration, and reasoning [33]. It uses ontologies to create a unified view of diverse data sources, enabling semantic interoperability. By establishing a common vocabulary and defining relationships among concepts, OBDI allows for the effective integration of data across domains. OBDI can help facilitate more accurate querying and analysis while also supporting reasoning capabilities to infer new knowledge based on the relationships defined in the ontology [34]. OBDI systems consist of three components: data sources, an OBDI ontology, and the mapping between them. The OBDI ontology defines the concepts and relationships in the shared data domain and may incorporate classes and relationships from existing ontologies. The mapping links the data sources to the ontology, using user-defined semantic relationships to link its terms and relationships, and then translates database content into triples. This forms a virtual knowledge graph that provides a cohesive user-defined semantic representation of the integrated data [35, 36].

In this paper, we describe the development of an OBDI workflow for the comparison of crop plant nutritional composition datasets. This study demonstrates concepts and technical methods needed to establish semantic relationships across various crop trait datasets relevant to a crop trait knowledge base. This involves semantic data integration applied to four heterogeneous publicly available nutritional datasets of UCs. Data were sourced from repositories that provide internally standardized and representative aggregate nutritional data for crops at the species or commodity level. We adopted the Minimum Information to Reference an External Ontology Term (MIREOT) approach, which allows the selection and reuse of well-established ontology terms that integrate standardized data description from the data source, ensuring semantic cohesion with existing plant science and associated domains. Data tables and fields were explicitly mapped to corresponding ontology classes or terms, enabling semantic alignment and consistent interpretation. We outline how this approach may facilitate data integration and assist in the wider adoption and utilization of UCs. We discuss how the integration of OBDI with machine learning can facilitate intelligent, accessible trait data management to enhance the adoption and advancement of artificial intelligence (AI)-driven decision-making.

## Materials and methods

### Source of data

Data were collated from primary and secondary sources (Table 1). Of these, CropBASE (https://cropbase.co.uk/cropbasev5/) houses data about the value chain and food systems for UCs, including production and costs. The crop-level nutritional concentration data were collected from the literature, which arose from various analytical methods and data presentations. To ensure consistency and minimize redundancy, we normalized units and categorical descriptors systematically across the entire dataset, resulting in a standardized and uniform representation of measurement units throughout the data. CropBASE adopts standards from the FoodData Central Database to control redundancies in the nutritional variables [37]. Joy *et al*. [38] published datasets of mineral composition of plant and soil samples collected from farmers’ fields and marketplaces in Malawi. The Food and Plant International (https://foodplantsinternational.com/plants/) provides nutritional information on a wide range of UCs, maintaining a consistent internal structure. However, it does not appear to follow any international standards for food classification and harmonization through cross-referencing external standards. The Priority Tree and Crop Food Composition databases [39] provide nutritional information about selected tree food crops, geographically focusing on Sub-Saharan Africa, to support their production and utilization. The data repository adopted the Food and Agriculture Organization’s International Network of Food Data Systems standard (www.fao.org/infoods/infoods/), providing a comprehensive framework for compiling, aggregating, and harmonizing food composition datasets.

**Table 1:** Nutritional components of UCs.

Data sources	Food types	Nutrients	References
Food composition database	132	32	[39]
Crops for the future/CropBase	528	112	[40]
Soil type influence crop mineral composition in Malawi	78	60	[41]
Priority food tree and crop	7771	8	https://foodplantsinternational.com/plants/

### Relational database design

A relational database schema was developed using concepts represented in the pooled heterogeneous datasets (Table 1), with 12 tables (Table 2) defined. The *crop* table represents the crops hosted in each database with a unique identifier that shows the source of that crop to preserve source-specific variations and maintain traceability. The *crop_part* table is a compiled anatomical entity for which the data were collected. The *experiment* table was created to denote the data repository where the datasets were collated, ensuring traceability of the dataset during query and retrieval. The *fao_classification* table models the soil classification of the location where the crop or soil sample was collected. The *food_group* table represents the food type, with the *location* table showing the sites where the samples were collected. The trait definitions are stored in the *trait_descriptor* table, while individual traits are catalogued in the *trait* table. The corresponding data values are stored in the *crop_score* and *soil_score* tables. Together, these tables form the data source providing structured access to the original trait data and supporting efficient querying and analysis.

**Table 2:** Relational database schema showing the table names, primary, and foreign key relationships.

Relations	Primary key	Foreign key
crop	crops_id	fao_classification_id, experiment_id
crop_part	crop_part_id	food_group_id
crop_score	crop_scrore_id	crop_id, trait_descriptor_id, trait_id
experiment	experiment_id	none
fao_classification	fao_classification_id	none
food_group	food_group_id	none
location	location_id	fao_classification_id, soil_type_id
soil_sample	soil_sample_id	soils_type_id
soil_type	soils_type_id	none
soil_score	soil_score_id	soil_sample_id
trait_descriptor	trait_descriptor_id	none
trait	traits_id	none

### Ontology annotation

In the context of each data source, metadata refers to information that describes the crops, crop parts, and traits captured in the *crop, crop_part*, and *trait* tables. The *crop* table was annotated with NCBI taxon terms, the *crop_part* was annotated with PO terms, and *trait* was annotated with either ChEBI, CDNO, FoodOn, Agronomy Ontology (AGRO), or National Cancer Institute Thesaurus (NCIT) terms. Manual annotation was performed based on core entities captured in the Investigation-Study-Assay framework that promotes structured metadata for research [19, 40] to enhance traceability and interoperability, making it appropriate for data integration.

### MIREOT-based ontology construction

**Figure 1:**
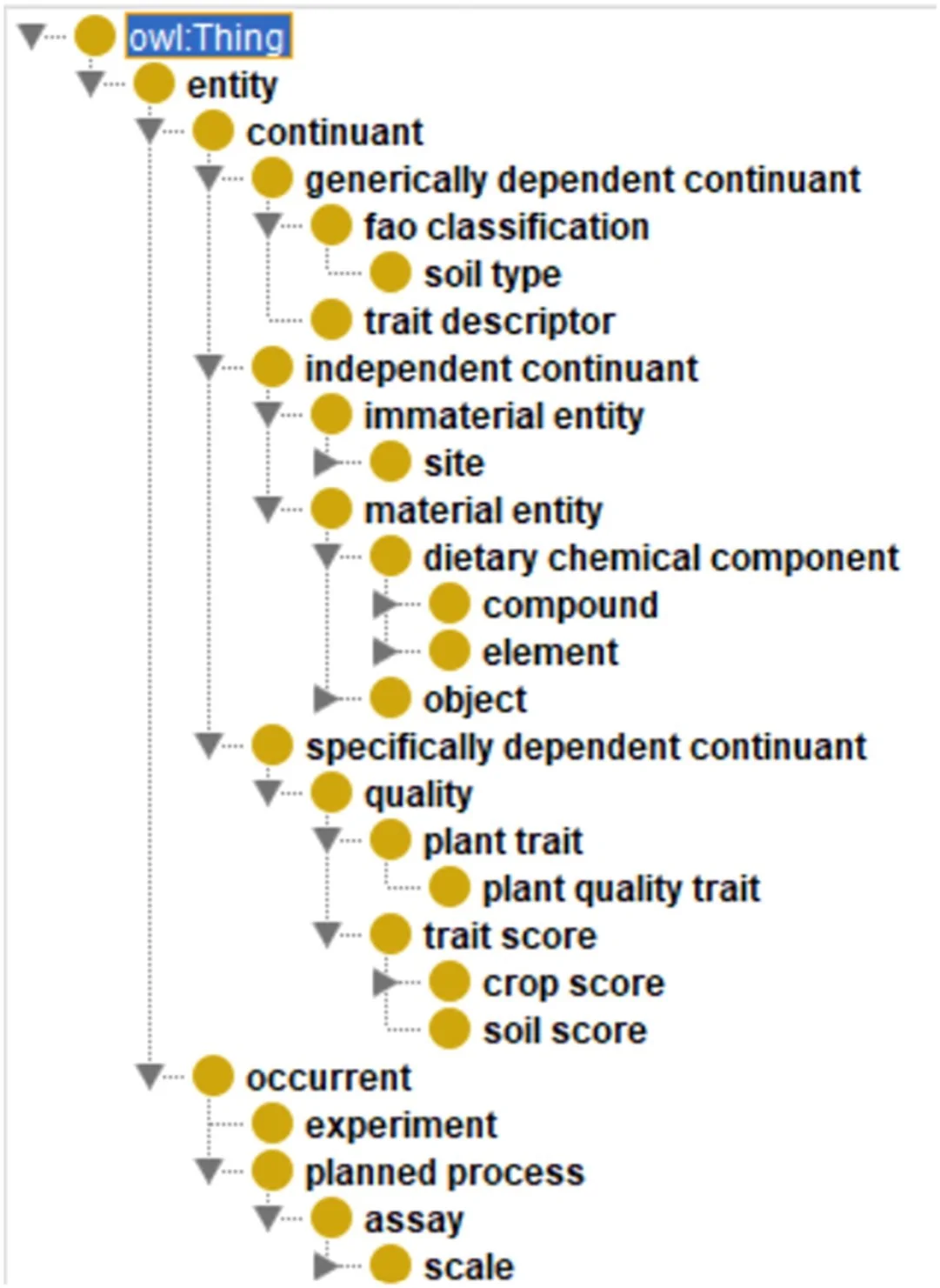
Crop Nutrition Application Ontology (CNAO) for UC nutritional data was constructed based on the class hierarchy and relation using Protégé. We used BFO as the upper-level ontology and imported terms from the OBO Foundry to develop a framework for data integration.

A MIREOT-based application approach ontology [41], ‘Crop Nutritional Application Ontology’ (CNAO), (https://github.com/AgnesAboagye/cnao/) was generated to represent data elements and the semantic relationships among them (Fig. 1). Protégé ontology editor version 5 was used to generate the ontology. Competency questions were formulated to serve as guidance in defining the scope, requirements, and validation of the ontology. These questions guided the formulation of specific queries that the ontology should be able to answer accurately and meet the needs of the intended domain and use cases. In addition to validating coverage, competency questions also informed the identification of essential classes and relationships required to enable semantic access to the underlying data sources [42]. Such competency questions included

1. What nutritional data are available for a specific crop across different experiments and locations?
2. What are the concentrations of essential nutrients (e.g. iron, calcium) in a specific crop from different sources?
3. Which crops have the highest nutritional value for a specific nutrient when grown in a particular location?

We used Basic Formal Ontology (BFO) [36] as the upper-level ontology as it is consistent with OBO ontologies and because it provides a framework for modelling relevant classes and properties and classifying them as a unified view. Furthermore, we reviewed entities within the existing ontologies, including PO, TO, AGRO, and CDNO, to find appropriate entities that can be reused within the MIREOT-based ontology. We examined the structure of the CGNO to ensure that it is comprehensive enough to represent accurately the wide variety of data from the different sources and meets the potential analysis needs of breeders and researchers.

### Mapping ontology to the data source

**Figure 2:**
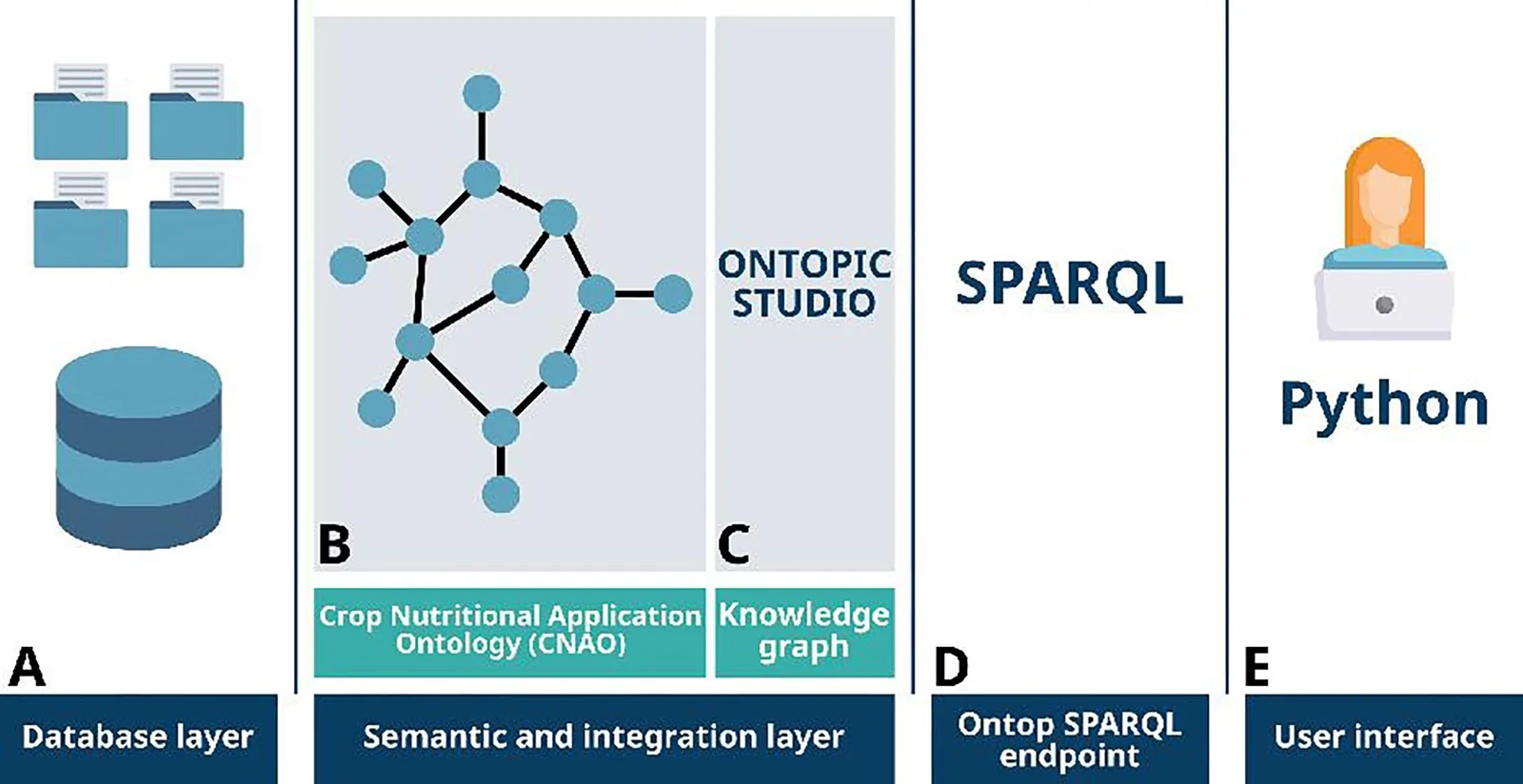
Overview of the semantic data integration framework aligned to the nutritional data of UCs. Even though developed with nutritional data of UCs, it is designed to be generic for all crops and centred around an ontology-based data access and integration (OBDI) approach. (A) The foundation of the framework lies in the database layer, which is made up of four heterogeneous datasets consolidated into a relational database serving as the system data source. The relational database was mapped into a unified conceptual representation, a MIREOT-based ontology known as Crop Nutritional Application Ontology (CNAO), to ensure semantic interoperability across the heterogeneous datasets. (B) CNAO captures domain knowledge and provides a shared vocabulary for describing entities such as crops, crop parts, traits, nutritional composition, and trait descriptors. (C) The underlying data is transformed into a virtual knowledge graph through mapping using the Ontopic Suite version 1.6, which defines how the tables and columns correspond to classes and properties in the MIREOT ontology (C). This forms a semantic and integration layer, which is a knowledge graph that can be queried using a SPARQL endpoint. (D) When a user (E) posts a SPARQL request against CNAO, the OBDI (A–D) framework semantically interprets the request and automatically translates the SPARQL query to SQL queries executed directly over the relational database. (E) Query results are visualized or transformed into other formats using Python libraries, creating a seamless approach to exploring, retrieving, and analysing data through an ontology-driven interface.

Ontopic Suite version 1.6 was used alongside the Java Database Connectivity Driver (JDBC) application programming interface to establish the connection to each original data source (Fig. 2A). For each table within the relevant data source, a corresponding virtual view was created. These virtual views enable the exploration and visualization of the content of the tables without altering the original data, enabling seamless integration with the ontology while preserving the integrity of the source data. Tables in the data source were mapped as classes, meaning that each row within the table, which is uniquely identified by a primary key, is an instance of the designated class. The associated columns (data fields) were mapped to data properties with explicitly defined data types such as date, string, or integer. In the MIREOT approach ontology, data properties serve as formal constructs that link individuals (instances of classes) to literal values, essential for representing measurable attributes as well as enabling meaningful querying and integration. Relationships between tables were mapped as object properties to link two entities together as subject and object. Data sources were mapped to the relevant components of the ontology, which are the classes and relationships in the ontology properties. This framework was generated to represent an RDF virtual knowledge graph enriched with semantic information from the upper-level ontology and relevant domain ontologies. The system therefore provides an ontology-based data access and integration (OBDI) that allows querying of the data using the elements in the ontology as predicates.

### Ontology reasoning and query

Through mapping, each data source is virtually exposed to the MIREOT-based ontology, utilizing the semantic information stored in the ontology to access data. To enable the efficient and meaningful retrieval of specific information from the data source. SPARQL queries were developed, ensuring that the queries could answer the original competency questions effectively and efficiently. Based on the mapping axioms defined for the MIREOT ontology, SPARQL queries are translated to equivalent structured query language (SQL) queries over the underlying relational database. These mapping axioms define the relationships between the ontology classes and terms and the relational database schema.

## Results

**Figure 3:**
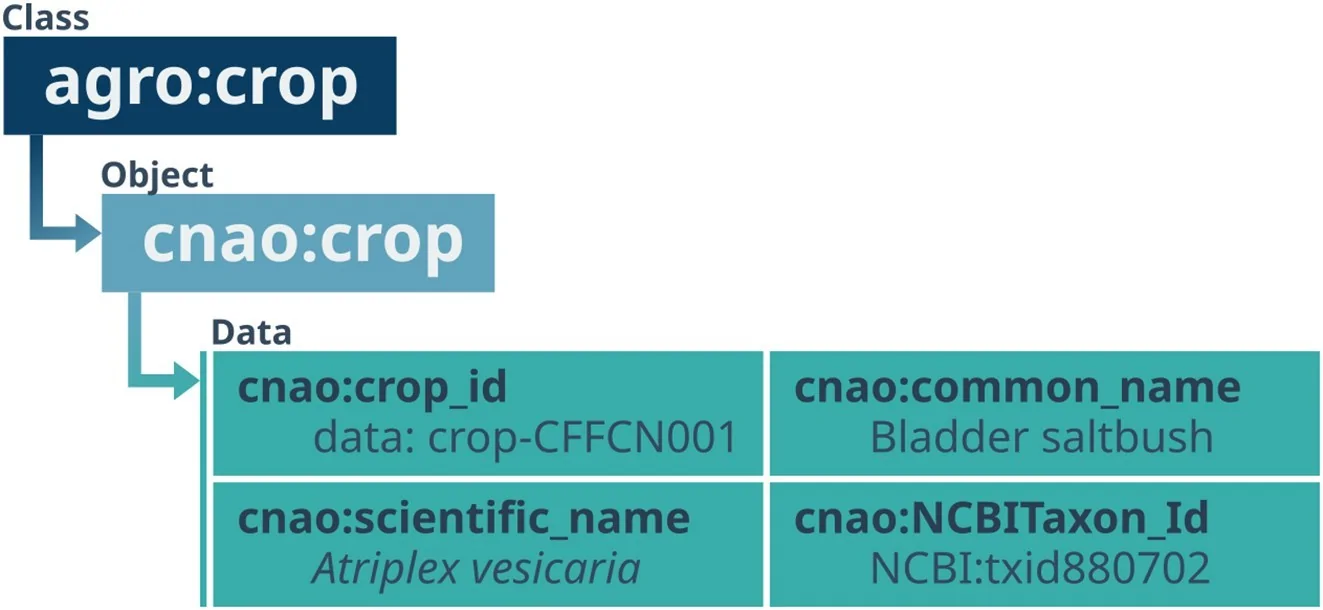
Development of domain knowledge graph in Ontopic Suite, showing semantic mapping of the database table ‘crop’ to the class agro: crop in the CNAO ontology, which was imported from the Agronomy Ontology (Fig. 2B). The mapping process is a core step in enabling ontology-based data access to relational data. Within the table, the column crop_id serves as the primary key. This is a unique identifier that is used to create a distinct instance (or individual) of the class agro: crop in the knowledge graph. This means that each row in the table corresponds to a separate crop entity in the ontology, uniquely identified by its crop_id. The other columns in the table, common_name, scientific_name, NCBITaxon_id, are mapped as data properties of the class agro: crop. These properties represent attributes and relations of each ‘crop’ instance, forming triples of the knowledge graph generated by the Ontopic Suite engine. These triples store the data in a human-interpretable format and enable the use of SPARQL queries.

Nutritional composition data were collated from four primary and secondary data sources for crop nutritional composition and processed to ensure syntactic and semantic uniformity. The format, nomenclature, measurement units, and completeness of the metadata varied within and between the source datasets. A systematic data wrangling process was therefore performed, including standardization of nutrient variable names, unit harmonization, and alignment of supporting metadata, such as analytical methods and data provenance. Manual curation was then used to eliminate missing values, duplication, and inconsistencies. Using this curated dataset, a normalized relational database optimized for ontology-based systems was generated. This guided the construction of the Crop Nutritional Application Ontology (CNAO) for annotation of UC nutritional data managed within an OBDI data model (Fig. 3). Developed in Protégé, the ontology combines pertinent domain ontologies from the OBO Foundry, such as the PO, Crop Dietary Nutrition Ontology (CDNO), TO, and NCI Thesaurus Agriculture (NCIT) and Agronomy Ontology AGRO), with the BFO as its upper-level foundation. New classes, object properties, and data properties were established to meet the predefined competency inquiries and analytical needs of researchers. The resulting model produced an RDF virtual knowledge graph that enables semantic querying through SPARQL using terms from the MIROET-based ontology. This semantic layer facilitates the retrieval of all instances associated with a specific class, encompassing its subclasses, thus enhancing query expressiveness and clarity. Compared to equivalent SQL queries, SPARQL queries generated through the ontology were found to be more concise and semantically meaningful, facilitating efficient access to integrated nutritional data.

### Example queries

A series of queries of varying complexity, based on the semantic connections captured by the CNAO, was generated to demonstrate the effectiveness of the OBDI framework, with crop nutritional trait datasets as a use case. The queries focus on the selection/filtering by crop, location, and trait to enable comparison of trait scores across the datasets.

#### Example 1: retrieving datasets relating to specific crops and their corresponding anatomical crop part (Fig. 4)

**Figure 4:**
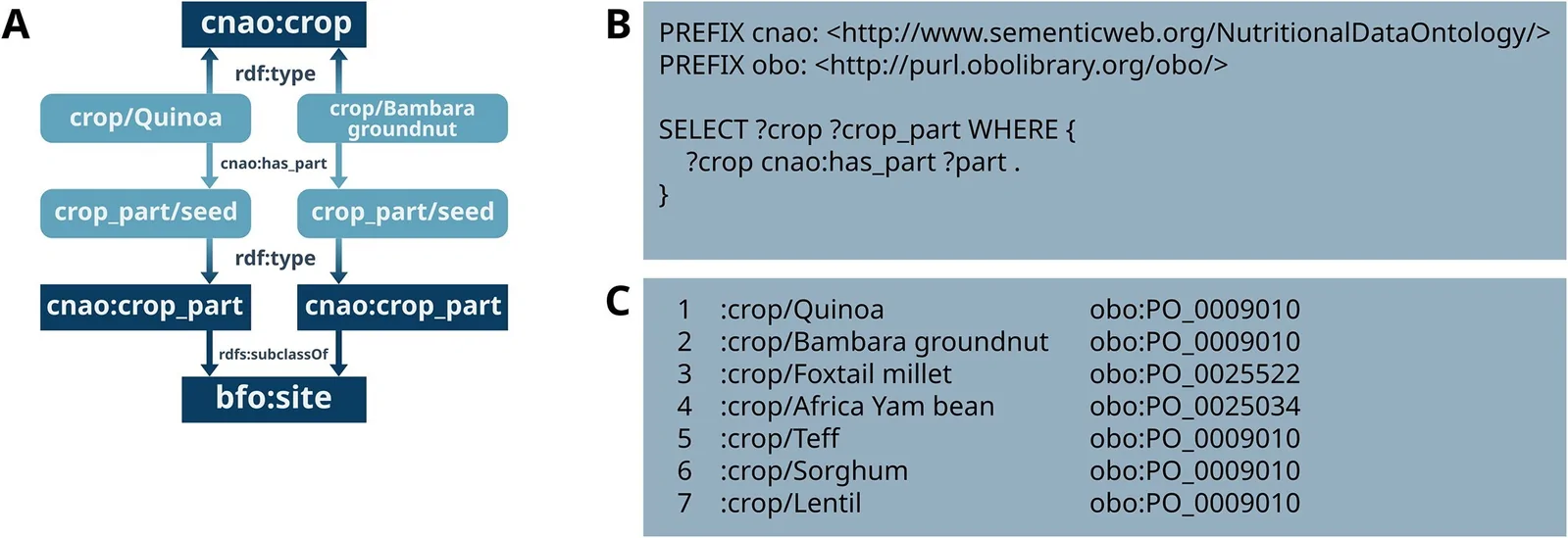
A graphical representation of the extraction of information where (A) is the semantic relationship between two classes (cnao: crop and cnao: crop_part) from the Crop Nutritional Application Ontology (CNAO): cnao: crop represents cultivated crops (bambara groundnut, quinoa) cnao: crop_part represents a specific anatomical part of a crop (tuber, seed). (B) SPARQL query that extracts all crops and their associated PO term (C) SPARQL query results.

The purpose of this query is to retrieve datasets related to specific crops and their anatomical parts by utilizing ontological relationships defined in the RDF knowledge graph. By using the object property *cnao: crop_part* from the CNAO, the ontology enables the linkage to the crop entities, and the corresponding *cnao: crop_part*, representing the anatomical entity. This object property enables a query to navigate the hierarchical or compositional relationship between a crop and its components, such as seeds, leaves, roots, or tubers. Using the ontology-based reasoning enhances the retrieval of implicit relationships defined in the CNAO, ensuring more complete and semantically meaningful results (Fig. 4).

#### Example 2: retrieving traits related to a specific crop (foxtail millet) (Fig. 5)

The query links ‘*crop*’ to ‘*traitName*’ to enable trait-based comparison and semantic integration. This query is designed to retrieve all traits associated with a specific crop (foxtail millet) in the semantically structured RDF knowledge graph and return the human-readable crop name and trait name. The query uses *obo: AGRO_00000325*, an RDF class representing individuals of the crop instance, to retrieve the crop name, with *cnao: has_trait* as the object property to navigate the trait names. Additionally, *cnao: traitName* and *cnao: cropCommonName* are used as data properties, providing a human-readable format for each trait name. The semantic relationship in the ontology correctly returns all traits associated with the specific crop. The outcome of the query is a sorted list of traits associated with the selected crop (in this case foxtail millet).

#### Example 3: retrieving trait scores of specific crops based on trait names and visualizing the results graphically (Fig. 6)

A query is designed to compare the trait scores for two specific crops (e.g. soybean and bambara groundnut) based on the trait names. SPARQL query was transformed to JavaScript Object Notation (JSON) format, processed into tabular format, and visualized. The query uses cnao: *cropCommonName* as a data property to provide a human-accessible crop name, *cnao: cropScore* as a numeric aggregate score for the given trait in the crop, *cnao: has_trait* as an object property that links the score to the trait, *cnao: traitName* provides a human-readable label for the trait, and *cnao: belongsTo* connects the traits to the experiments. The result of this query returns trait data records (scores) for the two specified crops. The results were further visualized graphically using the SPARQLWrapper Python library, which was used to interact with the SPARQL endpoint in Ontopic Suite, retrieving the data in a JSON format and subsequently transformed into structured data using the pandas Python library. Visualizations were generated using the GraphPad Prism version 10.6.1 to facilitate comparison and interpretation of trait values.

## Discussion

We have successfully demonstrated that an ontology-based data access and integration (OBDI) framework, utilizing a domain-defined general ontology (CGNO), improves the access, annotation, and comparison of nutritional data for UCs. Primarily, to demonstrate the importance of OBDI in the establishment of semantic relationships between diverse datasets that can be incorporated to operationalize a crop trait phenotyping knowledge base. The data workflow and knowledge graph (Fig. 2) enhance the semantic integration of heterogeneous data sources, thereby resolving semantic, schematic, and syntactic heterogeneity. This is significant because it is a common practice for different research groups or consortia to adopt their own format for storing and retrieving data [24, 43, 44]. Crop-specific standards for characterizing characteristics are frequently inadequately standardized across databases and efforts. For instance, both the Crop Ontology and the Plant Genetic Resources for Food and Agriculture descriptor lists offer practical frameworks for trait characterization, but they often employ distinct terminologies, structures, and granularity levels [24]. Such heterogeneity in data representation can often lead to challenges in data integration and interoperability, complicating the consolidation and comparison of datasets across investigations [24, 45]. Although the use case here was focused on nutritional data from UCs, the framework developed is designed to be generic for any crop and focuses on an ontology-based data access and integration (OBDI) approach. By using semantic web technologies (Fig. 2), including RDF and SPARQL, a consistent approach may be used to query and retrieve relevant information for downstream decision-making.

**Figure 5:**
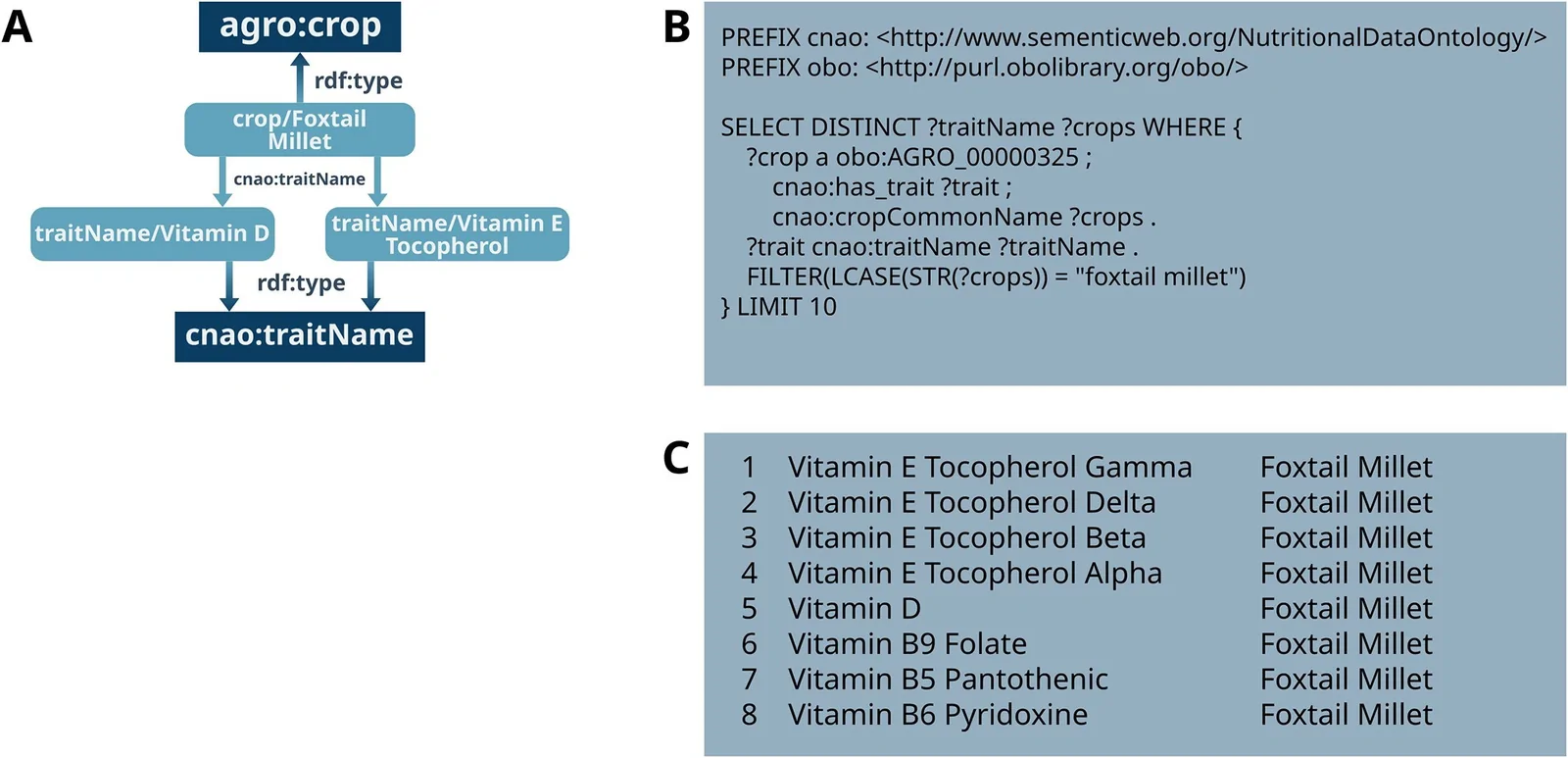
Graphical representation of the link between obo: AGRO_00000325 (in this case, foxtail millet) and cnao: trait, representing the associated phenotypic or agronomic traits. Each crop is identified using the data property cnao: cropCommonName, while each trait is described using the data property cnao: traitName, where (A) is the semantic relation between obo: AGRO_00000325 and the cano: trait, (B) is the SPARQL query, and (C) is the query output.

The CGNO ontology enhances data integration by using a shared controlled vocabulary that standardizes the meaning of the independently sourced data elements. This explicitly establishes their equivalence and facilitates inter-dataset comparison. For instance, the OBDI enhances the implicit relationships defined within the CNAO by using the attributes and relations defined within the MIREOT-based ontology (Fig. 4). This ensures semantically enriched insights, creating connections between related entities and revealing hidden patterns to facilitate a more thorough query answering and ultimately supporting more informed decision-making in plant nutrition, breeding, and crop improvement. Human-interpretable formats result from the ontology-based reasoning and data integration (Fig. 5), where complex semantic links, relations, and attributes are translated to formats that can be interpreted by non-technical users. This has the benefit of enhancing accessibility and promoting information exchange across disciplines and providing decision-making support.

The integration of aggregated values from multiple sources provides an overview of trait distribution between crops, which is significant in informing public health nutritional strategies, dietary recommendations, and food policy frameworks [3, 16]. Although breeders traditionally focus on yield, disease resistance, and climate adaptability, there is a growing demand for nutrient-dense and locally adaptable crops as resources for climate change and food and nutrition security [5]. Hence, they can also serve as a useful benchmark for crop breeders and researchers to identify genetic trends, establish breeding goals, and prioritize nutritional qualities for comparison across crops. We also discuss the potential of integrating OBDI with machine learning to facilitate intelligent and accessible trait data management, thereby enhancing the adoption and development of AI-driven decision-making.

### Ontology as a tool for integrating heterogeneous data

Ontologies enable the meaning and relationships behind data elements to be better understood by revealing information that may not be consistently or clearly represented. Such hidden information is revealed by semantic modelling of relationships among the data elements and by applying semantic reasoning to locally infer new knowledge that was not directly stored in the data sources. In general, mapping specific meta and data fields onto common ontological terms helps ensure consistency, semantic interoperability, and accessibility, thus promoting the FAIR data principles and stewardship. Achieving interoperability across vast and heterogeneous agricultural and nutritional datasets remains a significant challenge, yet it is crucial for informed decision-making by stakeholders, including farmers, breeders, researchers, and policymakers [46]. Despite their diversity, these datasets are highly valuable for enhancing agricultural practices, driving research innovation, and supporting evidence-based policy and management decisions. The data integration system here was established by loading the datasets into a single database as the data source and mapping these data sources to a common ontology (CNAO), thereby providing a unified view. This approach follows suggestions from [35, 43, 47] where a global-as-view ontology was created to integrate heterogeneous data for the analysis of research data and ensures a consistent, scalable, and accurate data curation and management strategy that conforms to the FAIR approach. This approach is important for improving plant breeding efforts [11] and overcoming the challenges of fragmented agricultural data, as it enables researchers and policymakers to access and analyse crop environmental information in a unified view [48]. The dynamic translation of SPARQL queries into SQL queries also avoids the overhead associated with migrating, duplicating, or maintaining data into a triple store [49]. Our proposed framework also supports scalability [49], especially as the number of source datasets grow, by simply connecting the entities to the ontology elements without modifying the underlying database structure or model [36].

### Leveraging OBDI for data comparison

**Figure 6:**
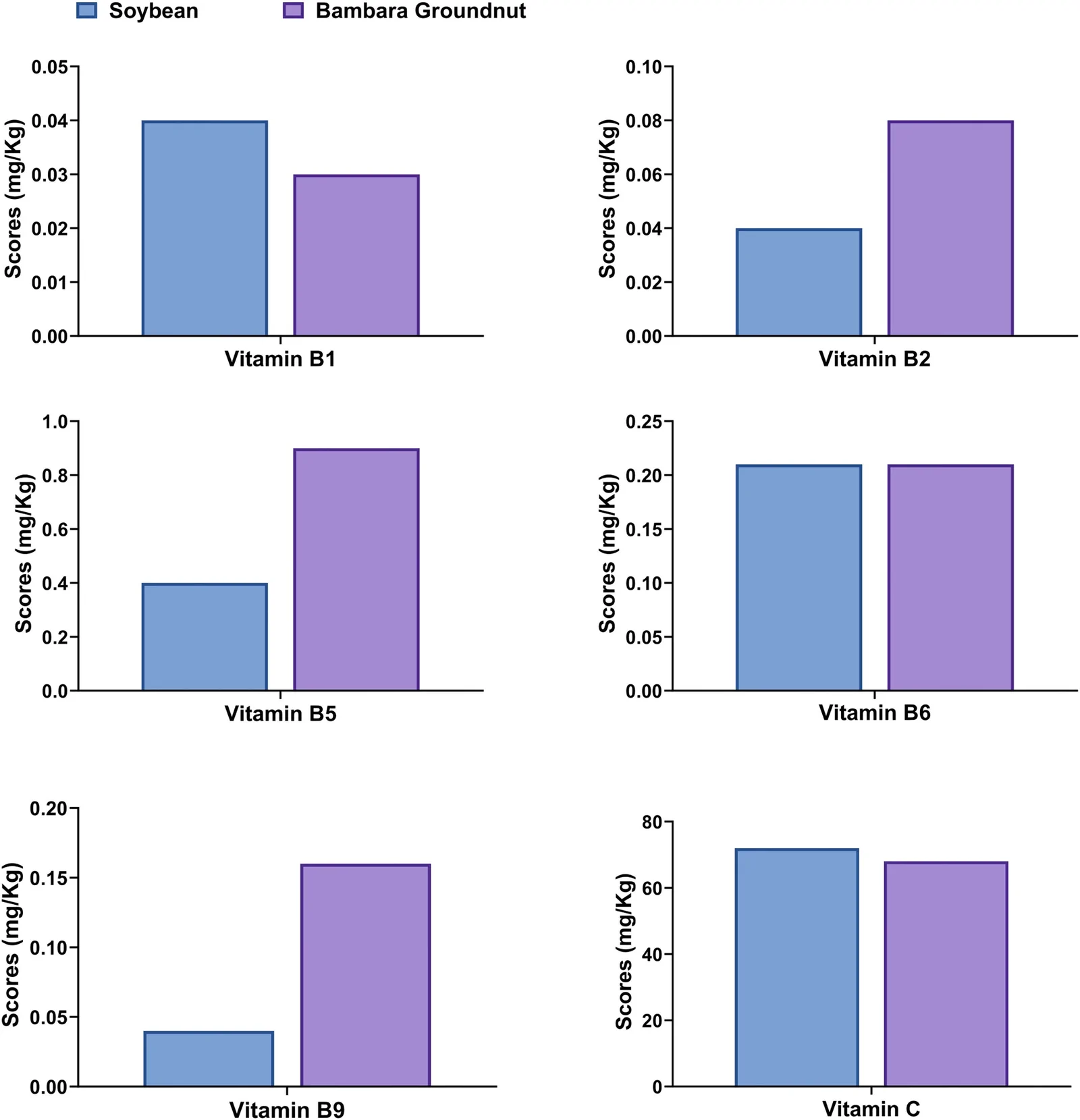
Comparison of the performance of soybean and bambara groundnut across specific nutritional composition data (e.g. Vitamin B9, Vitamin C, Vitamin B2). The data were retrieved from the data source described in Table 1 through the CNAO semantic knowledge base, structured into a table format, and visualized to support analysis and comparison.

Comparative analysis of crop traits, such as nutritional composition, may often be limited due to the complexities and divergence of underlying data representations. This challenge can be mitigated by adopting ontology-based data annotation and integration, which enable querying, visualization, and comparison [50]. For example, the CNAO property cnao*: crop_part* explicitly links various crops to their anatomical part through the semantic framework and allows effective query and comparison of nutritional profiles across crops (Fig. 4). Moreover, this formal representation supports automated reasoning, allowing explicit relationships to be clearly defined within the ontology. Again, data that are associated with distinct crops are annotated by the common property *cnao: has_trait*, resulting in a human-interpretable trait name and trait score, enabling different nutritional components to be compared (Fig. 6).

Data comparison is central to decision-making scenarios in many domains, including healthcare, finance, and environmental science. For example, ontology-based data integration (OBDI) facilitates the comparison and validation of clinical data from various sources, improving data quality and consistency through explicit relationship definitions, and allowing federated queries to prevent data duplication [43, 47]. Semantic web technologies have demonstrated efficacy in data integration, standardization of language, and facilitation of seamless data communication across domains in both the healthcare and finance sectors [46, 51, 52]. The OBDI approach we have outlined here is suitable for accessing valuable information for the development of nutrient-dense crops for health-focused or market-driven crop varieties.

### Role of OBDI to support AI and machine learning

Innovations in information-based crop improvement strategies, such as genomic selection [53], precision agriculture [54], and high-throughput phenotyping [55], are extending the impact of conventional and molecular plant breeding [56] to meet the growing demand for global food and nutrition security [37]. Development and implementation of crop-generic ontologies to annotate and extract information from heterogeneous data sources will assist in the application of machine learning algorithms for identifying concept relationships and properties [57–59], and will help bridge the gap between crop production, food supply and health outcomes, for example, in the development of nutrient-dense crops [60, 61]. In this context, OBDI can help ensure that AI and machine learning models have access to accurate and well-structured data for analysis and may accelerate the delivery of customized dietary recommendations and personalized nutrition [62]. The value of integrating heterogeneous datasets is greatly enhanced where crop phenotypic data are recorded using standardized metadata protocols, including ‘Minimal Information About Plant Phenotyping Experiments’ and Breeding Application Programme Interface, and stored in compatible FAIR-compliant databases such as the Germinate system [12, 13, 63].

## Conclusions

We present an ontology-based approach to data access and integration for integrative data analysis and comparison of trait data for UCs, using nutritional composition as a conceptual demonstration. We propose the Crop Nutritional Application Ontology (CAGO) as a first step to standardize the description, systematic representation, and integration of nutritional composition information, demonstrated by applying to four heterogeneous data sources. This framework establishes a foundation for explicit knowledge representation that can support hypothesis development and complex biological queries, building on existing ontologies in plant science and the nutrition domain [22]. The current implementation is given as a proof of principle, and it is recognized that further development of the data model would require extension to address experimental and environmental conditions. Future development will include extending the ontology to incorporate these and other factors, enhancing query capabilities, and refining integration methods to enable more comprehensive analyses. By exploring the practical implementation of this strategy, we illustrate the importance of robust and efficient data management in improving access to trait data for UCs, thereby promoting their wider adoption and utilization.

## Data Availability

The Crop Nutrition Application Ontology (CNAO), the ontology mappings, associated metadata, and the datasets supporting this study are freely available through the project web portal at (https://github.com/AgnesAboagye/cnao/). The source code, ontology files, documentation, and example SPARQL queries described in this article are openly accessible to facilitate data reuse, interoperability, and reproducibility in accordance with the FAIR (Findable, Accessible, Interoperable, and Reusable) data principles.
